# Mechanical FBG-Based Sensor for Leak Detection in Pressurized Pipes: Design, Modal Tuning, and Validation

**DOI:** 10.3390/s25237260

**Published:** 2025-11-28

**Authors:** Beatriz Defez, Javier Madrigal, Salvador Sales, Jorge Gosalbez

**Affiliations:** 1 Centro de Investigación en Tecnologías Gráficas, Universitat Politècnica de València, 46022 Valencia, Spain; 2 Institute of Telecommunications and Multimedia Applications (iTEAM), Universitat Politècnica de València, 46022 Valencia, Spain; jamadmad@iteam.upv.es (J.M.); ssales@dcom.upv.es (S.S.); jorgocas@dcom.upv.es (J.G.)

**Keywords:** fiber bragg grating (FBG), mechanical sensor (MS), leak detection, pressurized pipeline, modal tuning, finite element analysis (FEA)

## Abstract

This study presents the design, modeling, and experimental validation of a frequency-tuned mechanical sensor (MS) integrating a fiber bragg grating (FBG) for the detection of leak-induced vibrations in pressurized steel pipelines. Unlike conventional bonded FBGs—which directly follow the local wall deformation—the proposed MS consists of a base-fiber-mass transducer geometrically tuned so that its natural frequencies coincide with the dominant vibration modes of the pipe in the 5–7 kHz range. A combined framework of finite element analysis (FEA), computational fluid dynamics (CFD), and laboratory measurements was developed to assess the coupling between the pipe and the sensor. Results show that the MS behaves as a selective mechanical amplifier, enhancing strain sensitivity and signal-to-noise ratio (SNR) by up to 15 dB compared to a directly bonded FBG. The workflow integrates modal tuning, an equivalent harmonic excitation derived from CFD-based pressure fields, and frequency–response validation, leading to a mechanically optimized FBG transducer capable of discriminating high-frequency leak signatures. The excellent agreement between the simulation and experiment confirms that geometric resonance coupling provides an effective route to amplify leak-induced strain, offering a compact, scalable, and high-sensitivity solution for vibration-based leak detection in industrial pipelines.

## 1. Introduction

Leak-induced behavior in pressurized pipelines has been extensively investigated through computational fluid dynamics (CFD), fluid-structure interaction (FSI), transient hydraulic modeling, and structural dynamics, showing that even small defects generate highly localized pressure gradients, turbulent jets, and deformation concentrations capable of exciting the natural vibration modes of the pipe wall [1,2,3,4,5]. Numerous studies demonstrate that leak morphology—including circular orifices, longitudinal cracks, elliptical openings, and corrosion-induced thinning—significantly influences the internal pressure field and the resulting vibrational response [6,7,8,9]. CFD and FSI simulations consistently reveal that leakage jets cause sharp velocity and pressure fluctuations near the orifice, amplifying local deformation and interacting strongly with wall-thinning and corrosion defects [10,11,12]. Transient formulations further show that extended or crack-type leaks can alter modal damping and smooth resonance peaks, while preserving dominant vibrational content in the 3–10 kHz band [13,14,15]. Experimental and numerical works on water, gas, and crude-oil pipelines corroborate that leak excitation reliably activates structural modes in the 5–8 kHz range—far less affected by environmental and flow-induced noise than low-frequency components—highlighting the need for sensing strategies capable of capturing these high-frequency signatures with robustness and modal selectivity [16,17,18,19,20].

Leakage in pressurized pipelines represents a major concern for water distribution and industrial fluid transport systems, leading to substantial economic losses, environmental damage, and structural deterioration if left undetected [21,22]. Over the past two decades, fiber bragg grating (FBG)–based sensing technology has emerged as one of the most powerful tools for structural health monitoring (SHM) of pipelines, offering high sensitivity, electromagnetic immunity, multiplexing capability, and long-term stability under harsh conditions [23,24,25]. FBGs can track axial strain and temperature along the fiber, enabling the detection of mechanical anomalies such as leak-induced vibrations or wall thinning through localized deformation signatures [23,24].

Directly bonded FBG sensors have been widely implemented for monitoring steel and composite pipelines [24,26,27]. Their simplicity and accuracy make them attractive for laboratory and field applications; however, their performance strongly depends on the local strain field at the bonding interface, and therefore on the uncertain relative position of the sensor with respect to supports and leak locations [28,29]. Moreover, the frequency response of a directly attached FBG is inherently tied to the natural modes of the pipe, making it sensitive to noise and poorly selective in the presence of complex vibrational fields [27,30].

When a leak occurs, the escaping fluid acts as a localized and broadband excitation capable of activating the structural modes of the pipe [24,28,29]. The amplitude and frequency content of this response depend on the orifice geometry, leak size, fluid velocity, and internal pressure [31,32]. Low-frequency components are frequently masked by ambient noise, while high-frequency vibrations (typically 3–10 kHz) remain spatially localized and exhibit superior leak detectability [29,33]. Recent studies confirm that sensing strategies targeting this high-frequency band significantly improve the discrimination of leak-induced signatures from operational fluctuations [24,29,33].

A directly bonded FBG behaves as a passive strain gauge, reproducing the deformation of the pipe surface without introducing additional dynamics. Its performance therefore depends on whether the grating is located at a modal node or antinode. In contrast, a mechanical FBG sensor (MS)—a compact base-fiber-mass transducer—can be tuned to exhibit internal resonances that match the frequencies excited by leaks [28,30,34]. Acting as a mechanical filter, the MS selectively amplifies the strain components of interest while attenuating others, increasing the signal-to-noise ratio and enabling detection of secondary resonances (for example around 6–8 kHz) that remain hidden in the response of a directly bonded sensor [29,30,35].

This study presents the design, modeling, and experimental validation of a frequency-tuned MS optimized for leak detection in pressurized steel pipes. The methodology combines CFD to characterize the pressure excitation generated by a leak and finite element analysis (FEA) to simulate the coupled pipe-sensor dynamics. Through this combined approach, the geometry of the mechanical transducer can be systematically adjusted to achieve modal coupling with the dominant structural modes of the pipe, maximizing dynamic strain transfer to the FBG and improving detection robustness under realistic operating conditions [25,29,33,34,35].

Several mechanically assisted FBG configurations have been proposed to improve strain transfer and vibration sensitivity in pipelines. Non-intrusive clamp-on or beam-type structures convert circumferential deformation into axial strain, allowing rapid installation and replacement in field conditions [28,29,36]. Pipe-fixture sensors convert radial diameter changes into measurable axial strain and have proven effective for leak detection and corrosion monitoring when appropriately stiffened [29,32,37]. Additional studies have integrated distributed FBG arrays with machine learning algorithms to classify leak signatures or structural anomalies [25,38], while high-order FBGs have been employed for corrosion monitoring through spectral-broadening phenomena [31]. Long-term field deployments confirm that FBG networks can operate reliably in civil and industrial infrastructures [26,27,39].

Despite these advances, explicit modal tuning—designing the transducer so that its natural frequencies coincide with those of the pipe—has received limited attention. Most previous studies emphasize quasi-static amplification, temperature compensation, or signal post-processing [23,24,25,28,29,38], but seldom address sensor–structure modal matching as a primary design objective. The present work fills this gap by demonstrating a mechanically tunable FBG transducer capable of aligning its internal resonances with the dominant vibration bands excited by leaks, maximizing strain amplification and enhancing detection sensitivity.

Complementary advances in FBG-based vibration sensing—including phase-shifted gratings, multi-axis architectures, and mechanically amplified designs—show substantial improvements in responsiveness [40,41,42], while recent hybrid approaches combining mechanical amplification with AI classifiers for acoustic leak detection highlight further opportunities for performance enhancement [43]. Broader research in pipeline SHM continues to expand the sensing landscape [44], but does not incorporate modal tuning of the transducer itself as a central design concept.

Herein, we introduce a frequency-tuned mechanical sensor (MS) comprising a rigid base, a pre-strained silica fiber with an embedded FBG, and a small central mass. By selecting the fiber length, the attached mass, and the mounting position, the MS is tuned to the pipe’s dominant modes. Placing the MS at a deformation antinode converts it into a mechanical amplifier, increasing the FBG’s dynamic strain readout without electronic gain. We provide a complete pipeline—modeling, design of experiments (DOE), theoretical justification, CFD-derived loading, harmonic analysis, and experimental validation—demonstrating consistent amplification and improved detectability in the 5–7 kHz band.

For clarity and reproducibility, the entire modeling and validation procedure followed a structured sequence:

- Modal analysis of the bare pipe to identify natural frequencies and the spatial distribution of antinodes and nodes.
- Design of Experiments (DOE) evaluating the influence of the fiber free length L and proof mass diameter D on the natural frequencies of the fiber–mass subsystem.
- Assembly of the coupled pipe–sensor FEM model, including bonded contact at the pipe-base interface, rigid joints at the fiber clamps, and a lumped mass at the fiber midpoint.
- Generation of the equivalent excitation using CFD-derived pressure fields to define a localized 0.1 MPa pressure patch at the leak position.
- Harmonic response simulations of both the directly bonded FBG (DS) and the mechanical sensor (MS) under identical excitation conditions.
- Computation of the strain amplification.
- Experimental validation comparing the simulated frequency response with measured spectrograms during controlled leak events.

This workflow establishes a systematic methodology for designing and validating mechanically tuned FBG-based sensors in vibrating structures.

## 2. Materials and Methods

### 2.1. Geometry

The evaluation setup consists of a ¾″ steel pipe segment connected to a hydraulic circuit that enables controlled pressurization and leak testing. The circuit is supplied from a water network, with flow regulated by an inlet valve, while the outlet valve controls the working pressure. The internal pressure is continuously monitored using an analog manometer, allowing stable and repeatable test conditions to be maintained. A controlled leakage is generated through a 1 mm diameter pinhole that can be manually opened or closed by means of a threaded plug.

The test specimen used for both experimental and numerical analyses is a steel pipe of 450 mm total length, with an inner diameter of 21.33 mm and a wall thickness of 2.84 mm. The leak orifice is located at coordinates (x, y, z) = (250 mm, −13.5 mm, 0 mm), corresponding to the lower half of the pipe wall. Both pipe ends are rigidly clamped to reproduce the boundary conditions of the laboratory setup used for modal and harmonic testing.

This fixed-fixed boundary condition was selected to ensure direct correspondence between the experimental and numerical configurations. Although actual pipelines may exhibit more flexible supports or mixed boundary conditions, the present setup provides a controlled reference that isolates the dynamic coupling between the pipe and the sensor. Because both the MS and DS are mounted under identical constraints, any deviation from real in-field conditions affects both configurations equally, allowing a reliable comparison of their relative strain amplification. Moreover, at the high-frequency range of interest (4–8 kHz), the vibration modes are mainly local and weakly dependent on distant boundary effects, making the fixed-fixed assumption an acceptable and conservative representation of typical short-span pipe sections between supports.

Two sensing configurations were evaluated:

- DS: an FBG directly bonded to the outer wall of the pipe, acting as a direct strain pickup; and
- MS: an FBG integrated into a mechanical transducer composed of a base-fiber-mass assembly designed to amplify local strain and enhance detection sensitivity.

### 2.2. Computational Tools and Numerical Implementation

**Figure 1:**
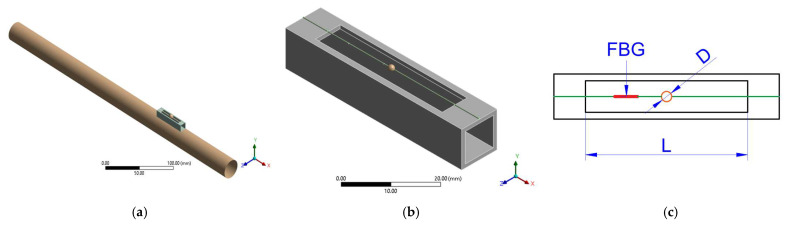
Graph of the steel pipe and the mechanical sensor (MS). (**a**) Coupled pipe-sensor model used in the finite element analysis; (**b**) isolated MS model showing the base-fiber-mass configuration; and (**c**) top view indicating the FBG position and the key design parameters: free fiber length (L) and proof mass diameter (D).

The pipe is made of steel, the sensor base is machined from aluminum, and the optical fiber is made of fused silica. Identical geometries, material properties, and mounting configurations were used in both the numerical and experimental setups to ensure direct correspondence between simulated and measured responses (Figure 1).

All numerical simulations were carried out using ANSYS Mechanical 2023 R2 (ANSYS Inc., Canonsburg, PA, USA) for the structural analyses, including modal, harmonic, and coupled pipe-sensor computations. The internal flow field used to derive the equivalent leak excitation was computed using ANSYS Fluent 2023 R2, ensuring consistency between the CFD pressure field and the structural loading applied in the harmonic simulations. These tools were selected because they allow for combining lightweight shell-beam structural models with CFD-derived localized excitations in a unified workflow.

The modal analyses were solved using the Block Lanczos eigenvalue extraction method, which provides stable and accurate results for lightly damped structures with well-separated natural frequencies. The harmonic response was computed using the full harmonic solver in ANSYS Mechanical under linear elastic and small-deformation assumptions, incorporating Rayleigh damping with a small mass-proportional coefficient to maintain numerical stability. The CFD simulations were performed using the segregated pressure-based solver in Fluent with second-order spatial discretization, enabling accurate characterization of the pressure gradients generated by the leak. All simulations assumed isotropic material behavior and steady-state fluid conditions.

All simulations were executed on a workstation equipped with an Intel Core i9-12900K processor, 64 GB of RAM, and an NVIDIA RTX A2000 GPU. While the structural solvers operate primarily on the CPU, documenting the hardware configuration ensures full reproducibility of the computational workflow and clarifies the resources required for the complete set of analyses.

The combination of Fluent for pressure-field estimation and Mechanical for structural dynamics was selected to address the specific requirements of this study: reproducing the localized excitation induced by a leak while maintaining a lightweight structural model capable of supporting extensive parametric and harmonic analyses. This integrated approach ensures that the numerical workflow remains both computationally efficient and physically representative of the experimental conditions.

### 2.3. Design Principles of the Mchanical Sensor

The MS was conceived as a mechanical amplification structure designed to enhance the strain transmitted to the FBG when the pipe wall is excited by external vibrations or internal pressure fluctuations. The operating principle is based on resonant amplification, in which the base–fiber–mass system behaves as a spring–mass oscillator tuned to coincide with the dominant vibration modes of the pipe. Near resonance, the relative motion between the proof mass and the base induces amplified axial strain in the fiber, substantially increasing sensitivity compared to the DS.

In this configuration, the fiber acts as the elastic element, the central proof mass provides the inertial load, and the aluminum base ensures both structural rigidity and efficient coupling with the pipe surface. By adjusting the geometric parameters—the proof mass diameter (D) and the free fiber length (L)—together with the mounting position, the MS can be tuned to target specific frequency bands associated with leak-induced vibrations. This design approach enables selective amplification of vibration-induced strains while maintaining the compactness and robustness required for practical field applications.

Two parameters govern the dynamic behavior of the MS: L and D. These variables determine, respectively, the stiffness and inertial properties of the system, and thus define its natural frequencies. The design strategy consists of selecting values of L and D so that the MS resonances coincide with the dominant modes of the pipe within the high-frequency band of interest (4–8 kHz). The choice of L is further constrained by the inter-nodal distance of the pipe’s vibration modes, ensuring that the sensor is mounted in a region of high modal deformation (antinodes) and that the fiber deforms efficiently without interference from nodal regions (Figure 1). This geometric–modal correspondence is essential to achieve optimal coupling between the MS and the host structure.

The base of the sensor provides mechanical stability and transmits dynamic energy from the pipe wall to the fiber. Its design is considered secondary relative to L and D, and is constrained to ensure that its own natural frequencies remain outside the target frequency band. This prevents the base from absorbing or diverting vibrational energy that should be transferred to the sensing element, guaranteeing efficient mechanical coupling and consistent strain amplification performance.

### 2.4. Meshing Strategy

A lightweight finite element model was developed to perform multiple design and validation analyses of the sensor-pipe system while keeping the computational cost low. The MS and the pipe were modeled primarily using surface and beam elements, an approach that preserves global stiffness and modal characteristics while significantly reducing the number of degrees of freedom. Although this simplification may slightly underestimate the overall stiffness, it provides an excellent compromise between accuracy and efficiency for both parametric and harmonic analyses.

The purpose of the model is not to achieve absolute numerical precision but to compare the relative performance of the DS and the MS. Therefore, the simplified representation is sufficient to capture the key physical mechanisms and the relative differences in strain amplification between configurations.

Both the steel pipe and the aluminum base of the MS were discretized using quadrilateral shell elements (Quad4). The mesh was generated with a Multizone method to ensure a structured distribution of elements along the axial and circumferential directions. The optical fiber containing the FBG section was represented as a line body with beam elements of 0.1 mm in length, totaling approximately 500 elements. This representation is particularly suitable because the beam’s axial strain directly corresponds to the strain experienced by the FBG, ensuring that the simulated response is physically consistent.

A preliminary mesh sensitivity study was performed to determine the optimal element size for each component. For the pipe, element sizes between 5 mm and 1 mm were tested; for the base, between 2 mm and 0.5 mm; and for the fiber, between 1 mm and 0.1 mm. The selected configuration (1 mm for the pipe, 0.5 mm for the base, and 0.1 mm for the fiber) showed the best convergence and frequency stability with acceptable computational time. The final mesh comprised 36,581 elements for the pipe and 6926 for the base. Mesh quality metrics (aspect ratio < 1.6, skewness < 0.5, and maximum corner angles < 120°) confirmed that all elements were within recommended limits, ensuring adequate accuracy for both modal and harmonic simulations.

To ensure full reproducibility of the computational experiments, the numerical model now explicitly defines all boundaries and contact conditions. Both pipe ends were constrained using fixed-fixed boundary conditions, enforcing zero displacement and rotation to reproduce the experimental setup. The aluminum base of the MS was connected to the external pipe wall through a bonded contact formulation based on an Augmented Lagrange method, ensuring full load transfer without relative slip. The FBG was represented as a beam line body rigidly attached to the base at both anchoring points using fixed joints, while the proof mass was implemented as a point mass coupled to the midpoint node of the fiber through a rigid body constraint. No frictional contacts or separation conditions were introduced, given that all elements are permanently bonded in the physical prototype. This set of assumptions guarantees consistent transmission of deformation from the pipe to the sensor and preserves the modal characteristics required for resonance tuning.

To reproduce the effect of the proof mass, a point mass was attached to the fiber at its midpoint, defined during the geometry modeling phase. This configuration allows the mass to act dynamically on the fiber, generating realistic bending and tensile effects near the midspan of the sensor. To represent the different functional regions of the fiber, the line body was divided into six segments: two terminal sections bonded to the base (representing the adhesive joints), two central segments with a midpoint vertex where the point mass is attached, and one intermediate segment on the downstream half of the fiber representing the FBG sensing region. Strain results obtained from this segment were used to evaluate and compare the axial deformation of the MS and the DS.

In the model, the FBG was represented as a beam element spanning the two anchoring points of the aluminum base, with a lead proof mass attached at its midpoint. This configuration replicates the actual experimental assembly, where the fiber is bonded to the base and the mass using small epoxy layers. In the physical prototype, the FBG was installed in a straight configuration under minimal manual tension, sufficient to maintain alignment and prevent sagging, without introducing measurable pre-stress. Consequently, the numerical model defined the FBG in its undeformed, straight state, fixed at both ends, which inherently represents the installed condition.

Introducing an explicit axial pre-tension in the model would artificially increase the stiffness of the fiber-mass assembly and shift the resonance frequencies upward without physical justification. Therefore, omitting explicit pre-tension ensures a more realistic dynamic behavior and maintains consistency with the experimental configuration.

In summary, the model assumes that the fiber is already in its operational straightened state, implicitly accounting for the small installation preload while avoiding unrealistic over-stiffening of the structure.

### 2.5. Material Behavior and Strength Considerations

All structural components in the numerical model were described using linear elastic and isotropic material behavior, consistent with the small dynamic strains generated by leak-induced vibrations. The steel pipe (AISI 1020), aluminum base (Al 6061-T6), and fused-silica fiber were assigned standard elastic properties (E = 210 GPa, 69 GPa, and 72 GPa; ν = 0.30, 0.33, and 0.17, respectively), ensuring accurate representation of stiffness and inertia in the dynamic response. Although the purpose of the simulations was not structural failure assessment, the maximum stresses predicted under the equivalent leak excitation were verified to remain well below the yield limits of all materials. Therefore, no nonlinear effects (plasticity, creep, or hyperelasticity) are expected, and the linear elastic formulation used in the modal and harmonic analyses is fully justified.

## 3. Results

### 3.1. Modal Deformation Analysis of the Pipe

The first numerical study consisted of a modal analysis of the pipe to identify its natural frequencies and mode shapes, particularly those above 3 kHz. This frequency range was selected because the MS is designed to detect high-frequency structural perturbations associated with leakage-induced vibrations. At lower frequencies, the response of the system is dominated by background noise sources such as pressure pulsations, pump or valve operation, mechanical vibrations from the mounting supports, and ambient noise from nearby equipment. These effects typically occur below 1 kHz and can mask the smaller, high-frequency vibrations generated by a leak.

The structural dynamics of the coupled pipe-sensor system were modeled assuming linear elastic behavior, small deformations, and steady-state harmonic excitation. The modal analysis solves the generalized eigenvalue problem

(K − ω2M)ϕ = 0(1)

where K and M are the global stiffness and mass matrices, ω is the natural circular frequency, and ϕ is the associated mode shape. The harmonic response analysis solves

(K + iωC − ω2M)u(ω) = F(ω)(2)

where C is the damping matrix (assumed Rayleigh-type with small damping ratios), u(ω) is the steady-state displacement field, and F(ω) is the equivalent load derived from the leak-induced pressure perturbation. This formulation provides the theoretical basis linking the modal tuning strategy to the frequency response predictions.

Focusing the sensor design on the higher-frequency modes of the pipe therefore enables a detection mechanism that is more selective and less sensitive to low-frequency disturbances, improving both signal-to-noise ratio and detection reliability.

**Figure 2:**
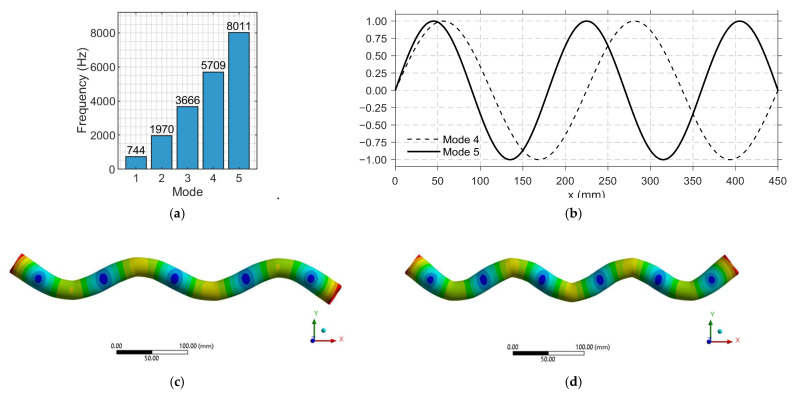
Modal analysis of the steel pipe. (**a**) Extracted natural frequencies within the 0–10 kHz range; (**b**) schematic representation of nodal and anti-nodal regions for vibration modes 4 and 5; (**c**) deformation pattern corresponding to mode 4 (≈5.7 kHz); and (**d**) deformation pattern corresponding to mode 5 (≈8.0 kHz).

The modal analysis identified the first five relevant vibration modes of the steel pipe within the 0–10 kHz range. The first three modes, appearing at 0.74 kHz, 1.97 kHz, and 3.67 kHz, correspond mainly to global bending and torsional deformations. These are strongly influenced by the boundary conditions and the overall geometry of the system and are not suitable for leak detection since they overlap with the low-frequency noise range (Figure 2a).

Above 5 kHz, the pipe exhibits higher-order flexural and circumferential modes at approximately 5.71 kHz and 8.01 kHz. These correspond to modes 4 and 5, respectively, which generate localized deformation patterns along the outer wall (Figure 2c,d). These higher modes are particularly relevant for sensing purposes because they produce measurable strain fields that can be effectively captured by the FBG sensors.

This modal characterization establishes the dynamic reference for tuning the parameters of the MS—specifically L and D—to achieve resonant coupling with the dominant vibration modes of the pipe in the high-frequency range (Figure 2b).

### 3.2. Design of Experiments (DOE) for MS Tuning

A DOE was conducted to evaluate the influence of L and D on the natural frequencies of the MS. In this analysis, only the fiber–mass assembly was modeled, excluding the aluminum base since its role is limited to providing structural support and transmitting dynamic energy from the pipe to the fiber without significantly affecting the sensor stiffness.

Parameter L represents the free span of the fiber between its anchoring points and directly governs the axial stiffness of the system. Longer fibers reduce stiffness and consequently lower the natural frequency, while shorter fibers result in stiffer behavior and higher resonant frequencies. The investigated lengths ranged from 30 mm to 40 mm, corresponding to the inter-nodal distances of the pipe’s higher-order modes identified in Section 3.1. This ensures that the fiber interacts efficiently with regions of maximum modal deformation (antinodes).

Parameter D defines the inertial contribution of the proof mass. Increasing D increases the total mass and therefore reduces the resonance frequency of the fiber–mass system. Three lead spheres with diameters of 1 mm, 1.5 mm, and 2 mm were analyzed, covering a practical range that allows tuning the MS resonance close to the dominant vibration modes of the pipe while maintaining compact dimensions and feasible manufacturing.

This parametric study provides the foundation for selecting the optimal MS geometry to achieve resonant coupling with the pipe and maximize strain transfer to the FBG sensing region.

The modal results obtained from the DOE reveal the combined influence of L and D on the dynamic behavior of the MS. For each configuration, the first five natural modes of the fiber-mass assembly were extracted, covering a frequency range from approximately 150 Hz to 12 kHz. The results confirm the expected opposite trends between stiffness and inertia: increasing L decreases the axial stiffness of the system, lowering the natural frequencies, whereas decreasing L shifts all modes upward. Conversely, increasing D introduces greater inertia and produces a corresponding reduction in resonance frequencies. These trends are consistent with the analytical response of a spring–mass oscillator and confirm the sensitivity of both parameters for resonance tuning.

Within the design range, the fundamental modes appear below 300 Hz and correspond primarily to global rigid-body motion of the mass and low-order bending of the fiber, which are not relevant for sensing purposes. The second to fourth modes, located between 3 kHz and 9 kHz, represent the first significant axial and flexural deformations of the fiber and are the most suitable for coupling with the vibration modes of the pipe identified in Section 3.1.

**Figure 3:**
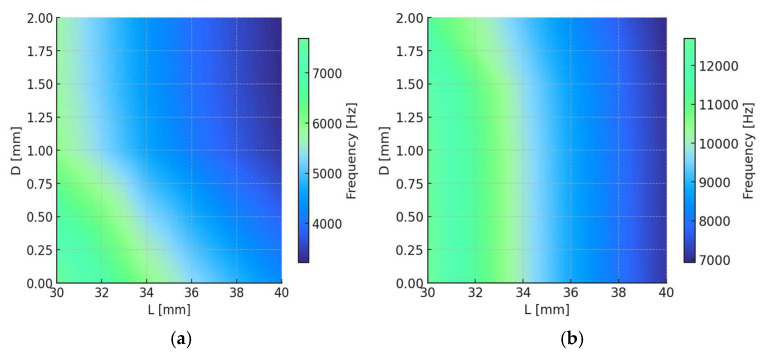
Interpolated heat maps of the natural frequency of the MS as a function of the free length L and the mass diameter D of the mechanical sensor: (**a**) Mode 4 and (**b**) Mode 5.

The analysis of modes 3 and 4 within the target frequency range shows a clear and monotonic dependence of the natural frequency on both geometric parameters. Increasing L reduces the system stiffness, leading to lower resonance frequencies, while increasing D increases inertia and produces a similar downward shift. These opposite but complementary effects define a two-dimensional design space where stiffness and inertia can be balanced to achieve resonance alignment with the dominant vibration modes of the pipe (Figure 3).

The analysis shows that for L = 36 mm, the modal distribution exhibits well-separated resonances, with a dominant deformation mode within the 4–9 kHz range that aligns with the main vibration modes of the pipe. Within this configuration, increasing D from 1 mm to 2 mm progressively shifts the principal resonance from approximately 4.1 kHz to 3.9 kHz, enhancing the potential for dynamic coupling and strain amplification.

Based on these results, the configuration L = 36 mm and D = 2 mm was selected for further analysis and experimental validation. This geometry offers the best compromise between frequency tuning, compactness, and mechanical amplification, ensuring resonance alignment with the dominant structural modes of the pipe while maintaining manufacturability and robust integration in the sensing setup.

**Figure 4:**
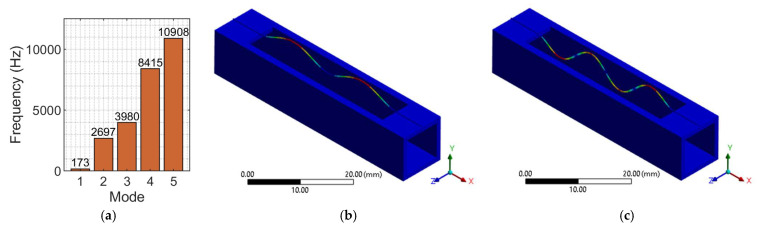
Modal analysis of the selected mechanical sensor (MS) configuration (L = 36 mm, D = 2 mm). (**a**) Extracted natural frequencies of the fiber-mass assembly; (**b**) deformation pattern corresponding to mode 3, characterized by a single axial strain lobe; and (**c**) deformation pattern of mode 4, showing multiple curvature reversals associated with higher-order dynamic amplification.

The third and fourth natural modes of this configuration, located at approximately 4.0 kHz and 8.4 kHz, respectively, fall within the target frequency band above 3 kHz and exhibit strong coupling with the fourth (≈5.7 kHz) and fifth (≈8.0 kHz) modes of the pipe. This spectral proximity, combined with the spatial coincidence of antinodes at the sensor location, enables efficient energy transfer and a measurable increase in strain amplification compared with the DS. Minor adjustments in the axial position or in D can be used to fine-tune this modal alignment when required (Figure 4).

### 3.3. Harmonic Response Analysis

A harmonic response analysis was performed to evaluate the dynamic strain behavior of the sensing system under periodic pressure excitations representative of a leakage event. This analysis provides the frequency-dependent response of both the pipe and the sensor, allowing identification of resonance conditions and estimation of the strain amplification achieved by the MS relative to the DS.

To define a realistic excitation load, a fluid–structure interaction (FSI) simulation was conducted using Fluent (steady-state) coupled with a static structural analysis. This study determined the pressure distribution exerted by the internal fluid on the pipe wall under both normal and leakage conditions. The results showed that the leak generates a localized pressure gradient in the region surrounding the orifice. From these data, an equivalent static pressure was derived to reproduce the same mechanical effect on the pipe in the structural model. The equivalent pressure was approximately 0.1 MPa, applied over a circular patch located at the same position and diameter as the actual leak. This load represents the pressure perturbation induced by the escaping fluid and was used as the excitation source for both numerical and experimental validation.

To reproduce realistic hydraulic conditions, the internal flow parameters used in both the CFD and experimental tests were defined according to the laboratory setup. The pipeline was filled with water at ambient temperature, with a dynamic viscosity of approximately 1 mPa·s and density of 998 kg·m^−3^. The internal pressure was maintained between 0.4 and 0.5 MPa, and the steady-state flow rate before leak activation was about 1.2 L·min^−1^. These values correspond to a turbulent but stable regime (Re ≈ 2.5 × 10^4^), representative of typical operating conditions in pressurized pipelines. Within this range, variations in viscosity or flow rate have a negligible influence on the modal frequencies of the pipe or on the relative strain amplification between the MS and DS. The mechanical amplification observed is governed primarily by the structural modal coupling between the pipe and the mechanical transducer, rather than by minor changes in fluid properties.

The leak was modeled as a localized pressure perturbation applied to the outer wall of the finite element pipe model at the exact coordinates of the orifice. The equivalent load was obtained from a steady-state CFD simulation of the internal pipe flow, in which a 1 mm diameter leak generated a concentrated pressure gradient around the orifice. The resulting pressure distribution was spatially averaged over the affected region to produce an equivalent circular pressure patch of 0.1 MPa used as harmonic excitation. This strategy avoids solving a full transient fluid–structure interaction (FSI) problem while preserving the spatial localization and intensity of the real leak-induced forces. The excitation is therefore a physically justified approximation that captures the dominant spectral features required for modal coupling with the MS.

Using this equivalent load, harmonic analyses were carried out on two finite element models: one including the DS and the other incorporating the MS. Both models were analyzed under identical boundary conditions and excitation parameters to enable direct comparison of their strain responses within the 3–10 kHz range. The sensors were mounted at the same axial position (x = 300 mm) on the pipe, a location identified in Section 3.1 as a region of high radial deformation amplitude (antinodes) for the dominant vibration modes. This placement ensures strong mechanical coupling between the pipe and the sensor.

This approach allows evaluation of the frequency-dependent strain transfer from the pipe wall to the FBG in both configurations, enabling quantitative assessment of the amplification provided by the MS and its suitability for leak detection in the high-frequency range.

A local mesh refinement was applied around the circular pressure patch to accurately capture the high strain gradients generated by the localized load. The smallest element size in this region was set to 0.2 mm, with several transition layers progressively coarsening toward the bulk mesh of the pipe. Additional mesh transitions were introduced between the pipe and the aluminum base to ensure smooth deformation transfer without artificial stiffness jumps. This refinement strategy ensured a stable and accurate harmonic solution while maintaining moderate computational cost.

The harmonic response analysis was also extended to four axial positions along the pipe—x = 275 mm, 300 mm, 325 mm, and 350 mm—to evaluate how local modal deformation influences the strain sensitivity of both sensors. These positions were selected based on the modal deformation profiles (Section 3.1), which reveal alternating nodes and antinodes of radial displacement along this region. Analyzing multiple locations enables a deeper understanding of the interaction between the sensor and the vibration field and verifies that the mechanical amplification effect remains effective even when the MS is not located exactly at a modal antinode.

**Figure 5:**
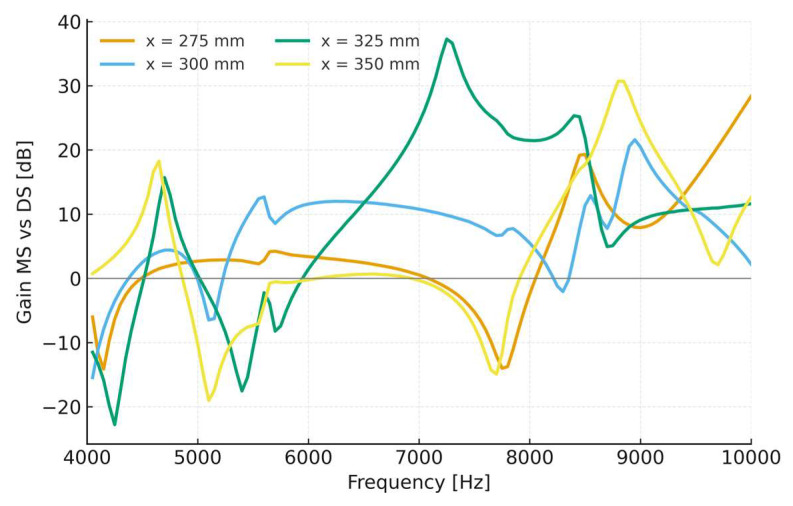
Figure response gain G(f) of the mechanical sensor (MS) relative to the directly bonded sensor (DS) at different axial positions along the pipe. The curves illustrate how the mounting position affects strain amplification, with the highest broadband gain obtained when the MS is located near a modal antinode (x = 300 mm).

For each axial position, the strain amplitude was computed for both configurations under the same equivalent excitation derived from the FSI analysis. The comparison was expressed as a strain gain, defined as G(f) = 20⋅lg(εMS/εDS) which represents the relative enhancement in strain response provided by the MS (Figure 5).

The results show clear differences among the four mounting positions along the pipe.

At x = 275 mm, the MS exhibits a steadily increasing gain with frequency, reaching values above +20 dB in the upper part of the spectrum (8–10 kHz). This behavior indicates strong amplification near the high-frequency resonances of the pipe, although sensitivity in the lower portion of the band (around 5–6 kHz) remains moderate.

At x = 300 mm, corresponding to a region of high modal curvature (antinode), the sensor displays a broadband enhancement, with gains of approximately +10 to +15 dB across most of the 5–9 kHz range. This configuration provides the most balanced response, combining effective amplification with stability throughout the target band (3–10 kHz).

At x = 325 mm, the amplification becomes frequency-selective, showing a strong resonance between 7 and 9 kHz (up to +20 dB) but a notable attenuation near 5.5 kHz. This pattern reflects the local transition from an antinode to a node in the pipe’s deformation field, where coupling efficiency temporarily decreases.

At x = 350 mm, the response is similar but slightly shifted toward higher frequencies, with limited gain below 7 kHz and recovery above 8 kHz, where the MS again outperforms the DS by more than +15 dB.

Overall, these results confirm that the axial position of the sensor strongly influences strain transfer from the pipe to the FBG. Positions close to antinodes maximize dynamic coupling and lead to broadband amplification, whereas locations near nodes reduce the response or shift the effective bandwidth. The x = 300 mm configuration stands out as the most robust, ensuring consistent sensitivity across the full high-frequency range (3–10 kHz) and validating the modal-based placement strategy for optimal sensor performance.

The observed variation of amplification with both frequency and axial position can be directly attributed to the coupling between the modal shapes of the pipe and those of the MS. Around 5 kHz, the response is dominated by the fourth vibration mode of the pipe, whereas between 7 and 8 kHz, the fifth mode becomes predominant. As the sensor moves along the pipe, its position alternately approaches or departs from the antinodes associated with these modes, producing significant differences in strain transfer efficiency. Additionally, small shifts in the MS resonance frequencies cause local amplification or attenuation, since different internal fiber–mass modes are excited depending on both excitation frequency and local pipe curvature. This combined effect explains the observed position-dependent and frequency-selective amplification behavior.

**Figure 6:**
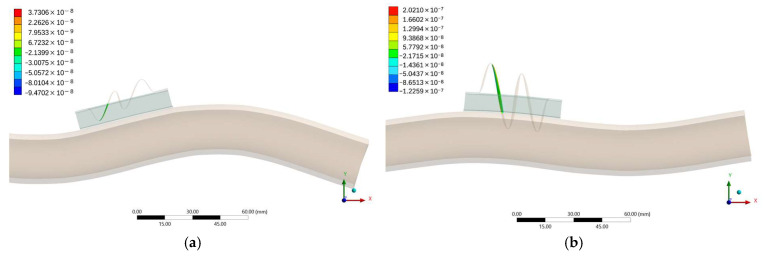
Longitudinal section of the mechanical sensor (MS) showing the deformation of the fiber under harmonic excitation: (**a**) at 5.6 kHz and (**b**) at 7.8 kHz. The longitudinal cut allows a clear visualization of the strain distribution along the fiber, and the position of the FBG sensing region is highlighted in green.

The deformation patterns obtained for the configuration at x = 300 mm were analyzed at two representative excitation frequencies. At approximately 5.6 kHz, the response is governed by the fourth vibration mode of the pipe and the third mode of the MS. In this condition, the fiber exhibits a low-order deformation pattern with a single axial strain lobe, promoting smooth and broadband amplification of the pipe-induced motion (Figure 6a). In contrast, at around 7.8 kHz, the excitation primarily involves the fifth mode of the pipe and the fourth mode of the MS, producing a higher-order deformation shape characterized by multiple curvature reversals and localized strain peaks near the proof mass and the downstream anchoring region (Figure 6b). This configuration corresponds to the activation of an internal MS resonance that strongly enhances the local axial strain and produces the high-gain region observed in the frequency–response curves.

The comparison between these two frequency cases confirms that the measured strain amplification originates from the combined modal coupling between the structural modes of the pipe and the intrinsic resonances of the MS. This interaction enables selective amplification of specific vibration bands—around 5 kHz and 7 kHz—corresponding to coupled mode pairs (pipe–MS), explaining the observed enhancement in sensitivity within the targeted high-frequency range.

## 4. Experimental Validation

**Figure 7:**
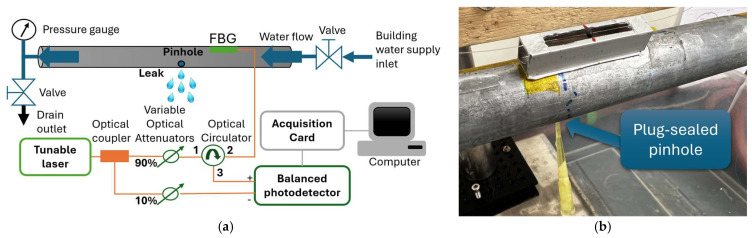
Experimental setup including the mechanical sensor (MS): (**a**) schematic diagram of the optical interrogation system used for FBG signal acquisition, and (**b**) photograph of the physical setup showing the MS mounted on the pressurized steel pipe under test.

To validate the numerical model and confirm the predicted strain amplification of the MS, experimental tests were conducted using the leak detection setup described in Section 2. The setup consisted of a steel pipe instrumented with FBG sensors and connected to an optical interrogation system for signal acquisition (Figure 7a). Measurements were performed sequentially: first, the DS was bonded to the outer wall of the pipe and tested; then, after its removal, the MS was installed at the same axial position (x = 300 mm) and tested under identical operating conditions. The leak was generated by manually opening a 1 mm pinhole in the pipe wall under controlled pressure, producing repeatable transient events corresponding to leak onset and closure (Figure 7b).

The FBG signals were acquired using an amplitude-based interrogation technique for high-speed dynamic measurements, in which spectral shifts are converted into variations of the reflected optical power. The relationship between these variations and the wavelength shift, and therefore the minimum detectable shift, depends on the spectral slope of the FBG at the laser wavelength. The higher the spectral slope, the lower the minimum detectable wavelength shift [40,43]. The wavelength shift was converted to strain using the typical strain sensitivity of 1.2 pm/µε for FBGs inscribed in standard optical single-mode fibers.

The laser wavelength was tuned to operate at maximum FBG slope point, providing an estimated wavelength resolution better than 1 pm, equivalent to a strain sensitivity below 0.8 µε. This resolution is sufficient to resolve the dynamic strain variations produced by leak-induced vibrations, with amplitudes of several microstrains, ensuring accurate strain representation in the frequency domain. Since this strain resolution is much lower than the measured strain amplitudes, the interrogator resolution has a negligible influence on the measured spectra or on the relative amplification observed between the mechanical sensor (MS) and the directly bonded FBG(DS).

**Figure 8:**
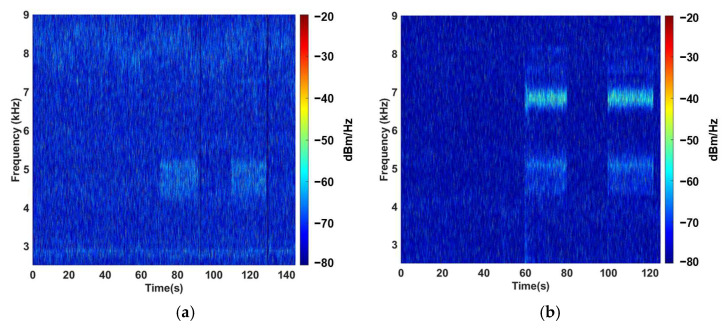
Experimental spectrograms obtained during leak testing: (**a**) directly bonded sensor (DS) and (**b**) mechanical sensor (MS). The MS exhibits enhanced spectral components around 5 kHz and 7 kHz, consistent with the dominant vibration modes of the pipe and demonstrating the strain amplification predicted by the harmonic analysis.

The frequency content of the recorded strain signals was analyzed using a short-time Fourier transform (STFT) to obtain the spectrograms of the DS and MS, both acquired under identical test conditions. The DS spectrogram displays low-amplitude components near 5 kHz during leak events, indicating a weak strain response to the vibrations induced by fluid escape. In contrast, the MS spectrogram reveals two well-defined spectral bands centered around 5 kHz and 7 kHz, corresponding to the dominant vibration modes of the pipe identified in the numerical modal analysis. These bands exhibit substantially higher power levels, confirming the strong mechanical amplification predicted by the harmonic simulations (Figure 8).

The experimental spectrograms revealed clear differences between the DS and MS responses. In the DS measurements, leak-induced activity appeared only as low-amplitude components near 5 kHz, with limited definition and poor separation from background noise. In contrast, the MS showed two well-defined spectral bands centered around 5 kHz and 7 kHz, consistent with the dominant vibration modes identified in the numerical modal analysis. These components appeared immediately after the leak was opened and disappeared after closure, confirming their physical origin. The MS response also exhibited significantly higher spectral energy, reflecting the strain amplification produced by the internal resonant modes of the base-fiber-mass system. The improved clarity and selectivity of the MS measurements demonstrate its ability to capture leak-induced high-frequency signatures that remain weak or partially masked in the DS recordings.

A direct comparison between the numerical predictions and the field measurements confirms the validity of the proposed FEM-based design methodology. The harmonic simulations predicted two amplification peaks within the 5–8 kHz range, corresponding to the fourth and fifth vibrational modes of the pipe and to the tuned resonances of the MS. These same frequency bands were observed experimentally in the MS spectrograms, with similar relative amplitude enhancement (approximately 10–15 dB). The DS measurements, both in simulation and experiment, displayed weaker strain levels and no clear higher-order features. The agreement between predicted and measured behavior demonstrates that the modal coupling mechanism is correctly captured by the numerical model and that the equivalent harmonic excitation derived from CFD provides a physically accurate representation of leak-induced loading.

The experimental results validate both the finite element model and the underlying design strategy of the MS. The enhanced frequency response of the sensor, particularly in the 5–8 kHz range, closely matches the simulated amplification behavior obtained from the harmonic analysis, where gains of 10–15 dB were predicted. The presence of identical spectral peaks in the experimental and numerical results confirms that the MS is effectively tuned to the resonant modes of the pipe and that the simplified shell–beam modeling approach provides a physically consistent representation of the real system.

Overall, the strong agreement between experimental and numerical data confirms the reliability of the FEM-based design methodology. The MS not only reproduces the expected resonant behavior but also significantly enhances the detectability of leak-induced vibrations compared with the DS, validating its suitability for high-frequency leak detection in pressurized pipelines.

## 5. Discussion

The results obtained in this work show that the mechanically tuned FBG sensor (MS) provides a clear enhancement in sensitivity and modal selectivity compared with the directly bonded FBG (DS), particularly in the 5–8 kHz band associated with leak-induced vibrations. This performance aligns with the well-documented fact that leakage events generate localized pressure gradients, turbulent jets, and wall deformation patterns whose dominant vibrational energy lies within the 3–10 kHz range [1,2,3,4,5,16,17,18,19,20]. Unlike conventional bonded FBGs—which rely exclusively on the local strain field and therefore inherit all uncertainties associated with sensor placement, modal nodes, or flow-induced noise—the MS introduces a tunable mechanical resonance that selectively amplifies the most informative vibrational components.

This sensing strategy differs substantially from the approaches proposed in earlier FBG-based pipeline monitoring studies, which typically focused on quasi-static strain transfer, direct pressure variations, or global deformation [23,26,34]. Early applications of FBG sensing, such as those presented by Jiang et al. [23], Li et al. [26], and Zhang et al. [34], mainly targeted low-frequency structural changes. While effective for long-term monitoring, these configurations are not optimized to capture high-frequency vibrational signatures arising from leakage. Similarly, distributed modal analysis approaches using FBGs in lightweight pipes [27] offer detailed information on global modes but do not incorporate mechanical amplification or resonance tuning.

Recent developments introduced non-intrusive strain-amplification structures, such as the sensitization mechanisms proposed by Yan et al. [28] and the high-sensitivity structures presented by Wu et al. [30]. Additional progress includes pipe-fixture-based FBG sensors for leak detection and corrosion monitoring [29,32,37], and high-order FBG-based corrosion sensors [31]. High-sensitivity optical designs such as phase-shifted gratings or balanced ultrasonic FBG systems [40] demonstrate substantial improvements in dynamic response, though they do not explicitly target modal alignment. Similarly, triaxial or enhanced-sensitivity FBG vibration sensors [41,42] increase responsiveness but remain fundamentally broadband and are not tuned to structural vibration patterns.

Several studies have explored leak detection via FBG vibration measurements. Kim et al. [24] reported distinct spectral components near 5–10 kHz during leakage, but their sensing configuration lacked resonance tuning and thus exhibited limited sensitivity. Other examples include UWFBG-array vibration sensing for gas pipelines [35] and quasi-distributed thermometric leak detection [37], which highlight the value of distributed sensing but do not incorporate mechanical resonance. Likewise, works using pipe-fixture FBG arrays [29,32] or strain-sensing methods for gas pipelines [38] emphasize pressure or strain-wave propagation rather than resonant coupling.

More recently, mechanically amplified FBG sensors have been combined with machine learning classifiers for acoustic leak detection in pipelines [43], revealing the potential of hybrid mechanical–AI approaches. Additional advances in pipeline monitoring architectures [44] continue to expand sensing capabilities but still do not integrate modal tuning as a primary design principle.

Compared with these methods, the proposed MS introduces a genuine resonance-based amplification mechanism: its internal modes (modes 3–4) are intentionally tuned to coincide with the dominant leak-excited modes of the pipe (modes 4–5). This modal alignment—supported by extensive CFD-FSI literature describing leak-excited high-frequency pipe modes [6,7,8,9,10,11,12,13,14,15]—explains the selective enhancement of the 5–8 kHz components observed numerically and experimentally in this study. To the best of our knowledge, this behavior has not been reported in previous pipeline FBG systems, which either operate in the quasi-static regime [26,28,31,34,40,41,42] or lack mechanical resonance tuning [23,24,27,29,30,32,35,37,38,43,44].

The strong numerical–experimental consistency demonstrated here further differentiates the proposed methodology. Prior works such as Altabey et al. [25] relied primarily on numerical SHM frameworks without experimental validation under real leakage conditions. Paolacci et al. [36] examined FBG-instrumented piping joints but did not explore resonance coupling. In contrast, the approximately 10–15 dB strain amplification measured in our experiments confirms the predictive capability of the reduced-order model, the validity of the modal tuning strategy, and the accuracy of the equivalent leak excitation derived from CFD—consistent with observations in recent high-fidelity leak simulations [8,12,14,19].

Moreover, the MS is non-intrusive, compact, and easily repositionable without damaging the pipe coating, unlike several bonded FBG approaches [28,32,34]. The internal resonant behavior also mitigates the strong dependence on sensor location that characterizes bonded FBGs. Even when installed at suboptimal positions or near modal nodes—where the DS response diminishes—the MS preserves detectable vibrational content through its own resonance amplification. This behavior parallels findings from distributed and crack-type leak studies, which show strong spatial variability in strain transfer and modal damping [13,14,15,18].

Overall, relative to existing FBG-based sensing approaches, the proposed MS provides:

- Resonance-based, frequency-selective amplification;
- Modal alignment with leak-excited modes;
- Non-intrusive and reconfigurable installation;
- Strong numerical–experimental agreement;
- Superior detection of high-frequency leak signatures that remain weak or masked when using directly bonded FBGs.

These advantages position the MS as a robust and complementary sensing strategy for high-frequency leak detection in pressurized pipelines and establish a complete CFD–FEM–experimental workflow for designing next-generation resonance-tuned optical sensors.

## 6. Conclusions

This study demonstrates that a frequency-tuned mechanical FBG sensor (MS), implemented as a base–fiber–mass transducer, can be systematically designed, modeled, and validated to detect leak-induced vibrations in pressurized steel pipelines with enhanced sensitivity relative to a directly bonded FBG (DS). The main conclusions are as follows:

A lightweight finite element modeling approach provides accurate and efficient predictive capability. The proposed shell–beam formulation, in which the pipe and MS base are modeled using shell elements and the fiber as a beam element with a lumped mass, successfully reproduces the coupled dynamics of the pipe–sensor assembly while maintaining low computational cost. Validation results show that this reduced-order model correctly captures resonance frequencies, relative strain levels, and amplification trends with higher consistency than a fully solid model, confirming its suitability for iterative design and modal tuning.

An equivalent excitation approach enables realistic harmonic validation without full transient FSI coupling. A steady-state CFD-based analysis was used to compute the pressure field associated with leakage, from which an equivalent static pressure patch of 0.1 MPa was applied at the leak location. This equivalent load reproduces the spectral characteristics of a real leak in the 4–8 kHz band, enabling accurate frequency-domain validation of the MS and reliable comparison with experimental measurements, while avoiding the computational cost of a fully coupled transient simulation.

The MS can be tuned to the 4–8 kHz frequency band, corresponding to the dominant vibration modes of the pipe. The DOE varying L (30–40 mm) and D (0–2 mm) revealed clear monotonic trends: increasing either parameter shifts the MS resonances downward. The resulting interpolated maps provide a quantitative basis for positioning the MS resonances inside the 4–8 kHz range, where leak-induced vibration energy is concentrated.

Geometric tuning results in measurable strain amplification. Configurations tuned within this band (for example, L ≈ 36 mm and D ≈ 2 mm) align MS resonances with the dominant pipe modes, yielding strain gains on the order of 10 dB (approximately threefold in amplitude or 15 dB in power). These amplification values are consistent with the numerical predictions and the experimental spectrograms obtained during controlled leak events. When installed at a modal antinode, the MS exhibits broadband strain amplification of approximately 10–15 dB across the target frequency range.

The axial position of the sensor is a critical design parameter. Modal analysis of the bare pipe is essential for identifying the distribution of axial nodes and antinodes. Mounting the MS at an antinode (for example, near x ≈ 300 mm in this setup) ensures efficient strain transfer and broadband amplification, whereas placement near a node leads to reduced coupling or narrowband response. This confirms the need to combine MS tuning with modal mapping of the host structure.

Experimental validation confirms the predictive accuracy of the numerical model. The measured spectrograms reproduce the spectral features predicted through FEM analysis, including the amplified components near 5 kHz and 7 kHz. The close agreement between the simulation and experiment validates both the mechanical tuning methodology and the equivalent harmonic excitation strategy.

The simplified modeling framework is sufficient for engineering applications. For practical leak detection, the relevant metric is the relative improvement of the MS with respect to the DS in the target frequency band. The shell–beam model with equivalent harmonic loading reliably predicts this relative response, enabling efficient parametric evaluation and optimization of L, D, and sensor placement—tasks that would otherwise require prohibitively expensive solid–fluid simulations.

### 6.1. Scope and Limitations

The current model assumes linear elastic behavior, fixed boundary conditions, and constant adhesive properties. Moderate deviations may arise from adhesive compliance, temperature-dependent pre-stress, or transient fluid effects; however, these influences can be assessed through sensitivity analyses or local mesh refinement without altering the main design conclusions.

### 6.2. Generalization and Implications

The workflow presented herein—modal analysis, DOE-based geometric tuning, equivalent harmonic validation, and experimental confirmation—constitutes a transferable methodology for designing mechanically selective FBG-based sensors in other vibrating structures or other frequency bands. Embedding spectral selectivity in the mechanical domain provides an inherently modal-matched transduction mechanism that complements traditional signal-processing strategies.

### 6.3. Practical Outcome

For leak detection in steel pipelines, the MS should be designed with its key resonances tuned within the 4–8 kHz band. Suitable dimensions include L in the range of 34–36 mm and D in the range of 1.5–2.0 mm. The sensor should be mounted at axial antinodes identified from modal analysis. Under these conditions, a strain amplification of approximately 10 dB relative to the DS can be expected, together with a stable broadband response covering the dominant leak-induced vibration range.

## 7. Future Research

Future research will extend the present design framework to explore alternative mechanical architectures for the sensor support, including optimized base geometries and lightweight topologies that enhance strain transfer and resonance amplification. Additional studies will analyze the influence of other geometric parameters—such as base curvature, adhesive thickness, and fiber anchoring configuration—on overall sensitivity and dynamic performance.

Further developments will include nonlinear and large-deformation modeling to capture the mechanical behavior of the MS under higher excitation levels or transient leak events. These advanced simulations will provide a more comprehensive understanding of the coupling mechanisms between the pipe and the sensor, particularly where the linear elastic assumption may no longer hold.

Moreover, experimental validation of alternative materials and manufacturing methods (e.g., additive manufacturing of the base or different proof-mass materials) will be pursued to improve robustness and facilitate industrial deployment. 

Future studies will also explore the influence of the working fluid on the sensor response. While the present validation employed water as the test liquid to ensure controlled and repeatable hydraulic conditions, extending the analysis to fluids with different viscosities and densities (e.g., oil-based or glycerin–water mixtures) will help quantify the effect of internal damping and excitation bandwidth on the dynamic coupling between the pipe and the mechanical sensor. This investigation will contribute to establishing fluid-independent calibration strategies and broadening the applicability of the proposed design to diverse industrial environments.

Finally, integrating the tuned MS into distributed FBG networks and evaluating its performance under realistic operating conditions—including temperature gradients and fluid–structure interactions—represent promising directions for future work.

## Data Availability

The raw data supporting the conclusions of this article will be made available by the authors upon request.

## Abbreviations

The following abbreviations are used in this manuscript:

C	Damping matrix (Rayleigh-type)
CFD	Computational fluid dynamics
D	Diameter of the proof mass
DOE	Design of experiments
DS	Direct sensor (directly bonded FBG)
FBG	Fiber bragg grating
F(ω)	Equivalent load derived from leak-induced pressure perturbation
FEM	Finite element method
FEA	Finite element analysis
K	Global stiffness matrix
L	Free fiber length
M	Global mass matrix
MS	Mechanical sensor (base–fiber–mass transducer)
ϕ	Mode shape vector
u(ω)	Steady-state displacement field
ω	Natural circular frequency
